# Supplementary material for: End Sequence Analysis Toolkit (ESAT) expands the extractable information from single-cell RNA-seq data
Source: Genome Res. 2016 Oct;26(10):1397–410. doi: 10.1101/gr.207902.116 (PMC5052061; doi:10.1101/gr.207902.116)
Supplement: Supplemental Material [file supp_gr.207902.116_Supp_Materials.tar.gz › ESAT-esat_v0.1/doc/html/interfacebroad_1_1core_1_1datastructures_1_1_interval.html]

ESAT: broad.core.datastructures.Interval Interface Reference


ESAT


- Main Page
- Related Pages
- Packages
- Classes
- Files

- Class List
- Class Index
- Class Hierarchy
- Class Members

All Classes Namespaces Files Functions Variables Enumerator Pages

- broad
- core
- datastructures
- Interval

Classes |
Public Member Functions |
Static Public Attributes |
List of all members

broad.core.datastructures.Interval Interface Reference

Inheritance diagram for broad.core.datastructures.Interval:

[legend]

|  |  |
| --- | --- |
| Classes | |
| class | **Impl** |
|  | |

|  |  |
| --- | --- |
| Public Member Functions | |
| int | getStart () |
|  | |
| int | getEnd () |
|  | |
| int | getLength () |
|  | |
| int | getRelationship (Interval interval) |
|  | |
| boolean | isAdjacent (Interval interval) |
|  | |

|  |  |
| --- | --- |
| Static Public Attributes | |
| static final int | HAS\_LESSER\_PART = 1 |
|  | |
| static final int | HAS\_OVERLAPPING\_PART = 2 |
|  | |
| static final int | HAS\_GREATER\_PART = 4 |
|  | |
| static final int | IS\_ADJACENT\_AND\_EMPTY = 0 |
|  | |
| static final int | IS\_STRICTLY\_LESS = HAS\_LESSER\_PART |
|  | |
| static final int | IS\_SUBSET = HAS\_OVERLAPPING\_PART |
|  | |
| static final int | IS\_LEFT\_OVERHANGING\_OVERLAPPER = HAS\_LESSER\_PART \| HAS\_OVERLAPPING\_PART |
|  | |
| static final int | IS\_STRICTLY\_GREATER = HAS\_GREATER\_PART |
|  | |
| static final int | IS\_RIGHT\_OVERHANGING\_OVERLAPPER = HAS\_GREATER\_PART \| HAS\_OVERLAPPING\_PART |
|  | |
| static final int | IS\_SUPERSET = HAS\_LESSER\_PART \| HAS\_OVERLAPPING\_PART \| HAS\_GREATER\_PART |
|  | |

## Detailed Description

Semi-open interval on the integer number line. Turf covered runs from the start value inclusive, up to, but not including, the end value.

Author
:   tsharpe

Version

Revision
:   74570

## Member Function Documentation

|  |  |  |  |  |
| --- | --- | --- | --- | --- |
| int broad.core.datastructures.Interval.getEnd | ( |  | ) |  |

Returns the ending point of the interval. The interval is not regarded as including this point.

Returns
:   The end.

Here is the caller graph for this function:

|  |  |  |  |  |
| --- | --- | --- | --- | --- |
| int broad.core.datastructures.Interval.getLength | ( |  | ) |  |

End - start.

|  |  |  |  |  |  |
| --- | --- | --- | --- | --- | --- |
| int broad.core.datastructures.Interval.getRelationship | ( | Interval | *interval* | ) |  |

Returns a constant that describes the relationship of this interval to a specified interval with regard to position on the number line.

Parameters
:   |  |  |
    | --- | --- |
    | interval | The interval to compare this one to. |

Returns
:   One of the IS\_\* constants defined above.

|  |  |  |  |  |
| --- | --- | --- | --- | --- |
| int broad.core.datastructures.Interval.getStart | ( |  | ) |  |

Returns the starting point of the interval.

Returns
:   The start.

Here is the caller graph for this function:

|  |  |  |  |  |  |
| --- | --- | --- | --- | --- | --- |
| boolean broad.core.datastructures.Interval.isAdjacent | ( | Interval | *interval* | ) |  |

Returns true if this interval ends where the specified interval starts, or vice versa.

Parameters
:   |  |  |
    | --- | --- |
    | interval | The interval to compare this one to. |

Returns
:   True, if adjacent.

## Member Data Documentation

final int broad.core.datastructures.Interval.HAS\_GREATER\_PART = 4

static

final int broad.core.datastructures.Interval.HAS\_LESSER\_PART = 1

static

final int broad.core.datastructures.Interval.HAS\_OVERLAPPING\_PART = 2

static

final int broad.core.datastructures.Interval.IS\_ADJACENT\_AND\_EMPTY = 0

static

final int broad.core.datastructures.Interval.IS\_LEFT\_OVERHANGING\_OVERLAPPER = HAS\_LESSER\_PART | HAS\_OVERLAPPING\_PART

static

final int broad.core.datastructures.Interval.IS\_RIGHT\_OVERHANGING\_OVERLAPPER = HAS\_GREATER\_PART | HAS\_OVERLAPPING\_PART

static

final int broad.core.datastructures.Interval.IS\_STRICTLY\_GREATER = HAS\_GREATER\_PART

static

final int broad.core.datastructures.Interval.IS\_STRICTLY\_LESS = HAS\_LESSER\_PART

static

final int broad.core.datastructures.Interval.IS\_SUBSET = HAS\_OVERLAPPING\_PART

static

final int broad.core.datastructures.Interval.IS\_SUPERSET = HAS\_LESSER\_PART | HAS\_OVERLAPPING\_PART | HAS\_GREATER\_PART

static

---

The documentation for this interface was generated from the following file:

- java/broad/core/datastructures/Interval.java


---

Generated on Fri Aug 15 2014 15:10:51 for ESAT by  

 1.8.7
